# Disentangling the mixed effects of soil management on microbial diversity and soil functions: A case study in vineyards

**DOI:** 10.1038/s41598-023-30338-z

**Published:** 2023-03-02

**Authors:** Martin Pingel, Annette Reineke, Ilona Leyer

**Affiliations:** 1 Department of Applied Ecology, Geisenheim University, Von-Lade-Str. 1, 65366 Geisenheim, Germany; 2 Department of Crop Protection, Geisenheim University, Von-Lade-Str. 1, 65366 Geisenheim, Germany

**Keywords:** Agroecology, Microbial ecology

## Abstract

Promoting soil functioning by maintaining soil microbial diversity and activity is central for sustainable agriculture. In viticulture, soil management often includes tillage, which poses a multifaceted disturbance to the soil environment and has direct and indirect effects on soil microbial diversity and soil functioning. However, the challenge of disentangling the effects of different soil management practices on soil microbial diversity and functioning has rarely been addressed. In this study, we investigated the effects of soil management on soil bacterial and fungal diversity as well as soil functions (soil respiration and decomposition) using a balanced experimental design with four soil management types in nine vineyards in Germany. Application of structural equation modelling enabled us to investigate the causal relationships of soil disturbance, vegetation cover, and plant richness on soil properties, microbial diversity, and soil functions. We could show that soil disturbance by tillage increased bacterial diversity but decreased fungal diversity. We identified a positive effect of plant diversity on bacterial diversity. Soil respiration showed a positive response to soil disturbance, while decomposition was negatively affected in highly disturbed soils via mediated effects of vegetation removal. Our results contribute to the understanding of direct and indirect effects of vineyard soil management on soil life and aids designing targeted recommendations for agricultural soil management.

## Introduction

Sustainable agricultural systems require the promotion of soil ecosystem functions. Soil bacteria and fungi are the main agents of soil functions like soil respiration, litter decomposition and carbon sequestration^1–3^. To guide decision making of farmers to support soil functioning, evidence about the linkages between soil microbial biodiversity, soil functions and soil management from in-field research is needed^4^.

In viticulture, tillage is the most important measure to control weeds besides herbicide application^5^. However, tillage represents a disturbance to the soil environment and is coupled with a number of undesired effects including alteration of the soil structure, reduction of aggregate stability, and long term depletion of soil organic carbon^6,7^. To circumvent the detrimental effects of tillage, vine-growers increasingly apply alternative soil management strategies including cover crops using grass species to facilitate soil stability or herbal seed mixtures to promote beneficial insects^8^.

Soil management measures also cause alterations of the aboveground vegetation including changes of vegetation cover density, plant species numbers and composition of the plant community and their traits. These multiple aboveground and belowground effects of soil management could directly or indirectly influence soil life including microbial diversity and soil functions. Studies in annual cropping systems^9,10^ as well as in perennial crops like grapevine^11,12^ found evidence for soil management effects on microbial diversity. However, these effects might depend on the time of sampling and the respective cultivar^11^, or might even vary between different years of sampling^13^. These findings put the generality of the effects of tillage on microbial diversity into question and raise demand for a detailed break-down of direct and indirect changes of the soil environment as a result of tillage.

For soil functions, it is generally assumed that undisturbed soils exhibit higher soil functioning rates than disturbed soils. Soil respiration has been shown to be lower in soils under tillage in comparison to no-tillage soils^6,14^. However, the incorporation of fresh plant residuals into the soil leads to an immediate flush of respiration activity and higher soil respiration rates compared to control treatments for up to 120 days following the tillage event^15^. For litter decomposition, there is evidence that decay rates of introduced litter, e.g. by litter bags, are lower in disturbed soils compared to less disturbed soils^12,16,17^.

For both microbial respiration and decomposition, a relationship between microbial diversity and rates of soil functions has been shown^18,19^. This raises the question whether the effects of soil disturbance on soil functions is solely due to abiotic changes in the soil or whether these effects are also indirectly mediated via alterations of microbial diversity^20^.

The challenge of disentangling and comparing the direct and indirect effects of soil disturbance by tillage on microbial diversity and soil functioning has rarely been addressed. There is a need for studies that apply harmonized experimental plots that allow comparing various soil management strategies on multiple fields. For this purpose, vineyards provide a good experimental system because different soil management types are applied, often within the same field. Additionally, vineyards as permanent cropping systems are considered as being more stable compared to annual cropping systems because the main crop is not changed annually^21^.

**Figure 1:**
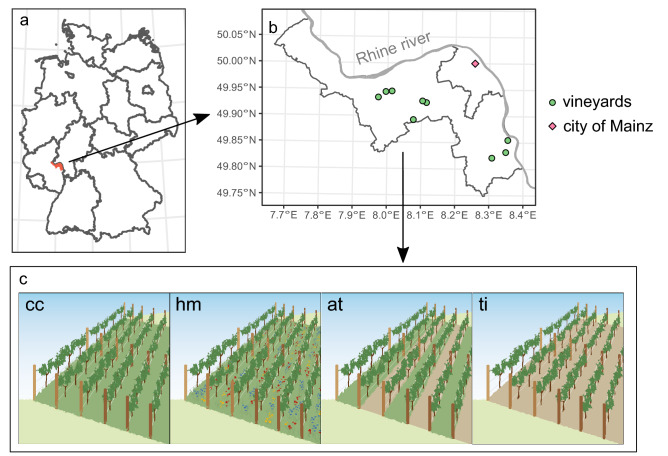
Location of experimental vineyards (**a**, **b**) and design of treatments (**c**). (**a**) Location of the study region (red shaded area) in Germany. (**b**) Experimental vineyards (green circles) were localized in the county ‘Mainz-Bingen’ close to the Rhine and the city of Mainz (purple diamond). (**c**) Illustration of soil treatments that were established in each experimental vineyard: ‘cc’: complete cover, ‘hm’: herbal mixture, ‘at’ alternating tillage, ‘ti’ tillage.

Here, we investigated the effects of vineyard soil management on bacterial and fungal diversity as well as soil functions (soil respiration and decomposition of two different substrates) using a balanced experimental design covering nine vineyards in Rhine-Hesse, Germany (Fig. 1a, b). In all vineyards, we established four experimental plots representing four soil treatment types (Fig. 1c): Tillage (ti): Vegetation was removed in all inter-rows by mechanical tillage twice a year; alternating tillage (at): Tillage in every second inter-row twice a year while in every other inter-row mulching was applied; herbal mixture (hm): Inter-rows were tilled at the beginning of the study followed by seeding a herbal seed mixture; complete cover (cc): Vegetation of all inter-rows (covered with grass-dominated vegetation) was managed by regular mulching depending on regrowth. These four treatment types represent a gradient of soil disturbance, which could be quantified by tillage frequency.

Our design enabled us to (i) investigate the effects of commonly used soil management practices on soil functions, soil microbial diversity, soil variables (soil organic carbon and total nitrogen), plant species richness and vegetation cover and to (ii) disentangle the causal linkages between these parameters using structural equation modeling. The outcome of this study could contribute to the development of targeted soil management options to maintain and promote soil functioning, which is pivotal to pave the way towards sustainable agriculture.

## Results

### Effects of soil treatment on microbial diversity, soil functions, and vegetation

First, we tested the effects of soil treatment types on bacterial and fungal Shannon diversity, soil functions (soil respiration, decomposition of green tea and roobios tea), vegetation cover, plant species richness, as well as soil organic carbon (OC) and soil total nitrogen (TN) using linear mixed models and analysis of variance (ANOVA). Numerical ANOVA results are given in Supplementary Table S1.

The microbial DNA sequence data included in total 6,931,404 reads for bacteria (ranging between 13,651 and 101,292 per sample) and 11,203,343 reads for fungi (ranging between 30,136 and 125,885). Reads were assigned to a total of 5739 bacterial OTUs (between 1923 and 4203 per sample) and 2688 fungal OTUs (between 428 and 1295 per sample). As a metric for bacterial and fungal diversity, we calculated the Shannon Diversity indices (H’) for both groups.

**Figure 2:**
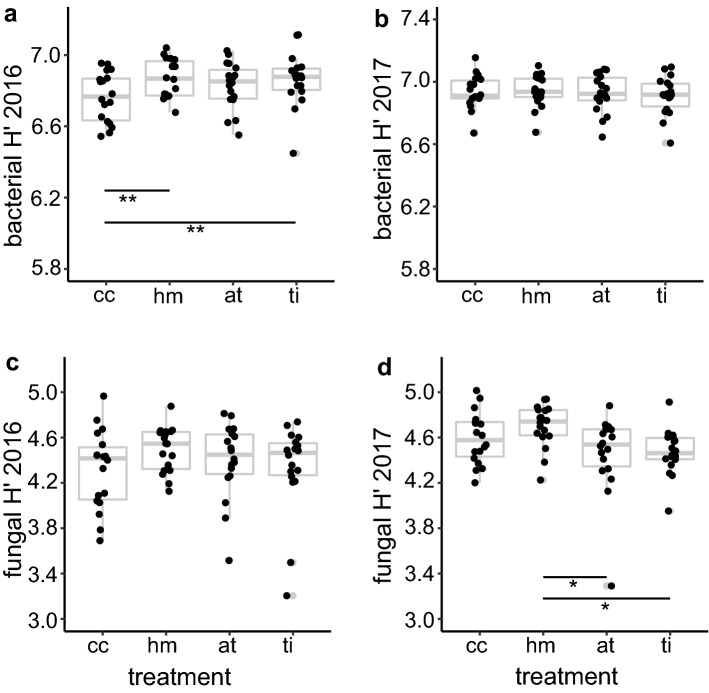
Response of vineyard soil microbial diversity to soil treatment types (‘cc’ complete cover, ‘hm’ herbal mixture, ‘at’ alternating tillage, ‘ti’ tillage). (**a, b**) Soil bacterial Shannon diversity (H’) in 2016 (**a**) and 2017 (**b**). (**c, d**) Soil fungal Shannon diversity (H’) in 2016 (**c**) and 2017 (**d**). Data points are represented by black dots; 25% and 75% percentile, mean, and range of data are represented by grey box-whisker plots. Horizontal bars indicate significant differences between treatments at levels *p* < 0.05 (*), *p* < 0.01 (**).

Soil bacterial diversity showed a significant response to soil treatment in 2016 (F = 5.92, *p* = 0.0013), but not in 2017 (F = 0.58, *p* = 0.6304). Bacterial diversity was lowest for the ‘cc’ treatment and significantly different from the ‘ti’ and ‘hm’ treatment (Fig. 2a). This pattern was not reflected in 2017 (Fig. 2b). Soil fungal diversity showed a significant response to soil treatment in 2017 (F = 4.24, *p* = 0.0088), where it was significantly higher for the ‘hm’ treatment compared to ‘at’ and ‘ti’ treatments (Fig. 2d). This trend was not significant for 2016 samples (Fig. 2c).

**Figure 3:**
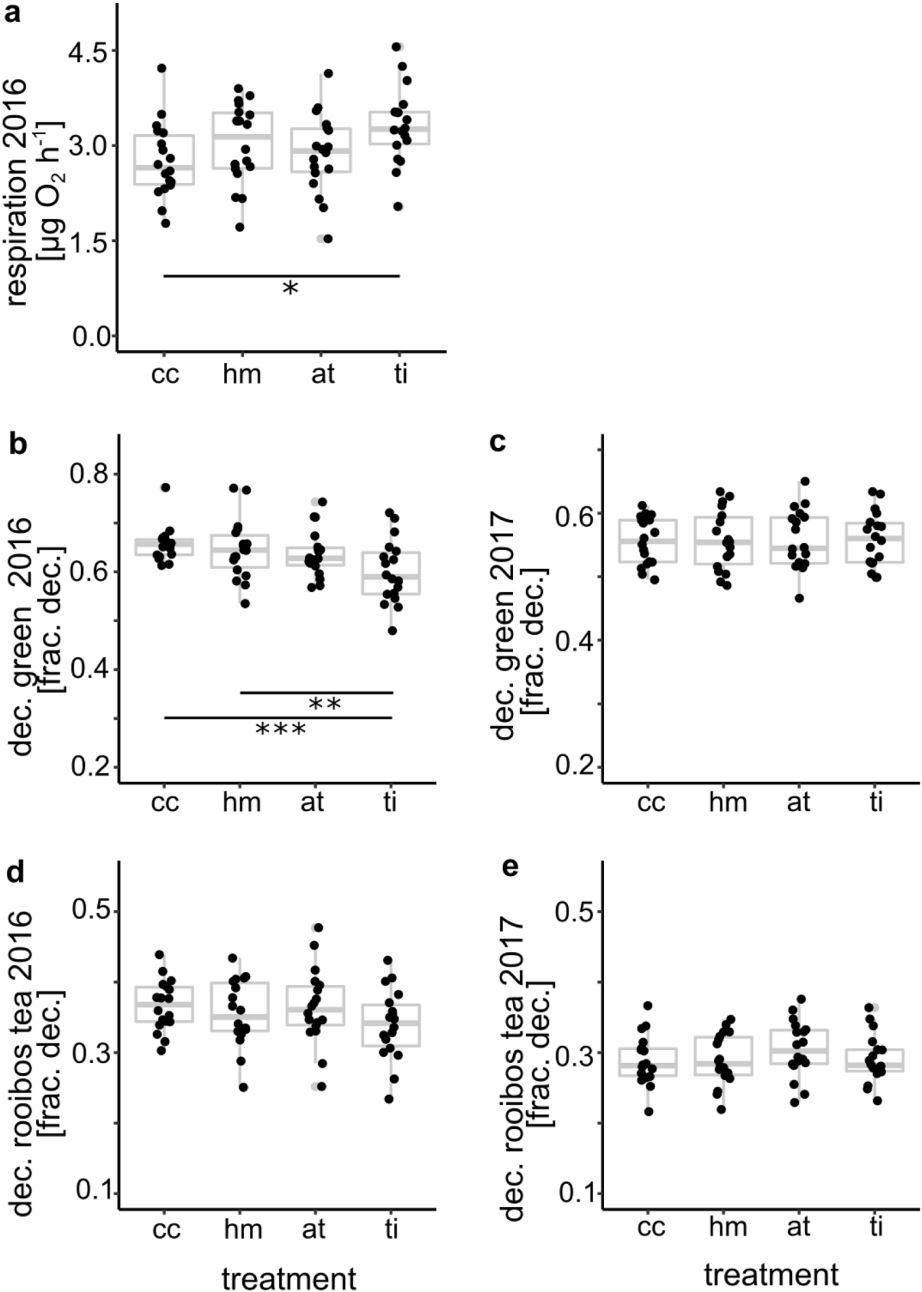
Response of soil functions to soil treatment types (‘cc’ complete cover, ‘hm’ herbal mixture, ‘at’ alternating tillage, ‘ti’ tillage). (**a**) soil respiration 2016, (**b, c**) decomposition of green tea 2016 (**b**) and 2017 (**c**), (**d, e**) decomposition of rooibos tea 2016 (**d**) and 2017 (**e**). Data points are represented by black dots; 25% and 75% percentile, mean, and range of data are represented by grey box-whisker plots. Horizontal bars indicate significant differences between treatments at levels *p* < 0.05 (*), *p* < 0.01 (**), *p* < 0.001 (***).

Soil respiration, which was only measured in 2016, was significantly higher for the ‘ti’ treatment than for the ‘cc’ treatment (Fig. 3a, F = 3.56, *p* = 0.0194). Decomposition of green tea, which represents labile organic material, showed a significant response for the 2016 data (F = 7.03, *p* = 0.0001), where the decomposed fraction was significantly lower for the ‘ti’ treatment compared to the ‘cc’ and ‘hm’ treatment (Fig. 3b). This pattern was not resembled for 2017 data (Fig. 3c, F = 0.04, *p* = 0.9892). Decomposition of rooibos tea, which represents recalcitrant organic material, showed no significant response to soil treatment in 2016 (Fig. 3d, F = 2.42, *p* = 0.0748) or 2017 (Fig. 3e, F = 1.52, *p* = 0.2172).

**Figure 4:**
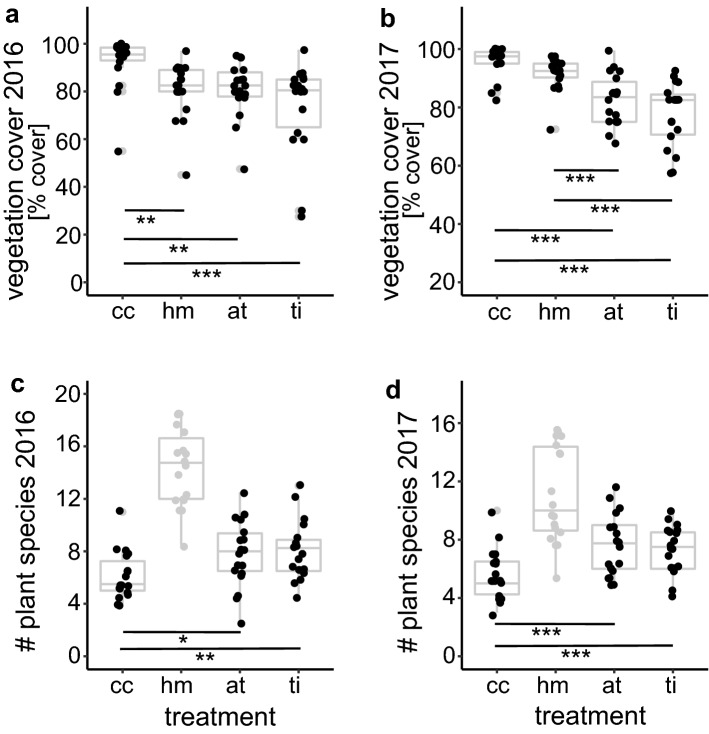
Response of vegetation variables to soil treatment types (‘cc’ complete cover, ‘hm’ herbal mixture, ‘at’ alternating tillage, ‘ti’ tillage). (**a, b**) vegetation cover 2016 (**a**) and 2017 (**b**). (**c, d**) plant species richness 2016 (**c**) and 2017 (**d**). Data points are represented by black dots; 25% and 75% percentile, mean, and range of data are represented by grey box-whisker plots. Horizontal bars indicate significant differences between treatments at levels *p* < 0.05 (*), *p* < 0.01 (**), *p* < 0.001 (***). For plant species richness, treatment ‘hm’ was not included in regression analysis, because of experimental manipulation of species richness in these plots.

Vegetation cover and plant species richness showed significant responses to soil treatment for both sampling years (2016: F = 12.55, *p* = 0.0001; 2017: F = 28.19, *p* = 0.0001). For 2016, vegetation cover was highest for the ‘cc’ treatment and was significantly different from the other treatments (Fig. 4a). For 2017, vegetation cover was significantly higher for ‘cc’ and ‘hm’ treatments than for ‘ti’ and ‘at’ treatments (Fig. 4b). For both years, plant species richness was highest for the ‘hm’ treatment (Fig. 4c, d). However, since plant species richness in these plots were manipulated by sowing and not induced by soil disturbance, it was not included into the statistical analysis. For the treatment types besides ‘hm’, plant species richness was higher for ‘ti’ and ‘at’ treatments compared to the ‘cc’ treatment for both years (2016: F = 7.09, *p* = 0.0022; 2017: F = 14.88, *p* = 0.0001). There was no significant effect of soil treatment on soil OC and TN content for 2016 (OC: F = 0.39, *p* = 0.7608; TN: F = 0.50, *p* = 0.6861) or 2017 (OC: F = 0.94, *p* = 0.4277; TN: F = 0.60, *p* = 0.6184).

### Structural equation modeling

**Figure 5:**
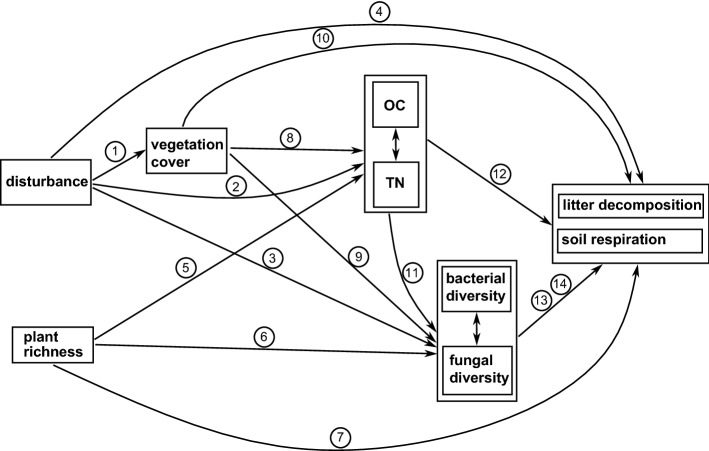
Meta model depicting hypothetical causal relations between variables soil disturbance, plant species richness, vegetation cover, soil organic carbon (OC) and soil total nitrogen (TN), soil microbial diversity (Shannon diversity), and soil functions (soil respiration and litter decomposition). Arrows show direction of causal relation. Double headed arrows indicate relationships modelled as bi-directional effects (correlations). For references and sign of the hypothetical effect, see Supplementary Table S2.

To unravel the direct and indirect effects connecting soil disturbance, vegetation, soil variables, soil microbial diversity, and soil functions, we applied structural equation models (SEM). The advantage of SEM is their capability to unite multiple predictor and response variables in one causal network and to disentangle direct and indirect effects. This requires an a priori theoretical path model (meta model) based on informed hypotheses about causal relationships between variables. We developed such a meta model (Fig. 5, Supplementary Table S2) and derived candidate models that could be fitted using soil respiration, decomposition of green tea, and decomposition of rooibos tea as terminal response variable for each sampling year (2016 and 2017) and for both years combined. In the following we focus on SEM paths with significant standardized coefficients (at level *p* < 0.05). All path coefficients and statistics are given in Supplementary Table S3.

### Vegetation cover and soil variables

**Figure 6:**
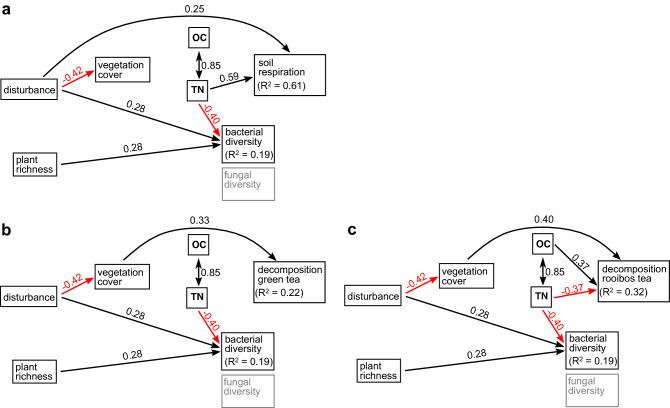
Structural equation models for the sampling year 2016. Subfigures show results for different soil functions as terminal response variable: soil respiration (**a**), decomposition of green tea (**b**), decomposition of rooibos tea (**c**). Arrows show direction of significant effects at *p* < 0.05, line color indicates sign of relationship (black: positive, red: negative). Double-headed arrows indicate bi-directional correlation. Standardized coefficients are given as numbers next to the arrows. Marginal R^2^-values of soil functions are given for microbial diversity and soil functions. Variables with no significant pathway are shaded in grey.

**Figure 7:**
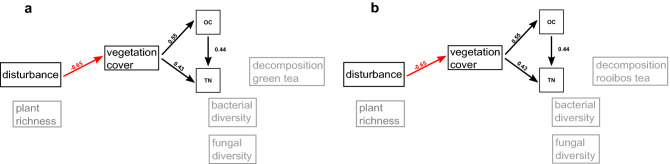
Structural equation models for the sampling year 2017. Subfigures show results for different soil functions as terminal response variable: decomposition of green tea (**a**), decomposition of rooibos tea (**b**). Arrows show direction of significant effects at *p* < 0.05, line color indicates sign of relationship (black: positive, red: negative). Double-headed arrows indicate bi-directional correlation. Standardized coefficients are given as numbers next to the arrows. Marginal R^2^-values of soil functions are given for microbial diversity and soil functions. Variables with no significant pathway are shaded in grey.

**Figure 8:**
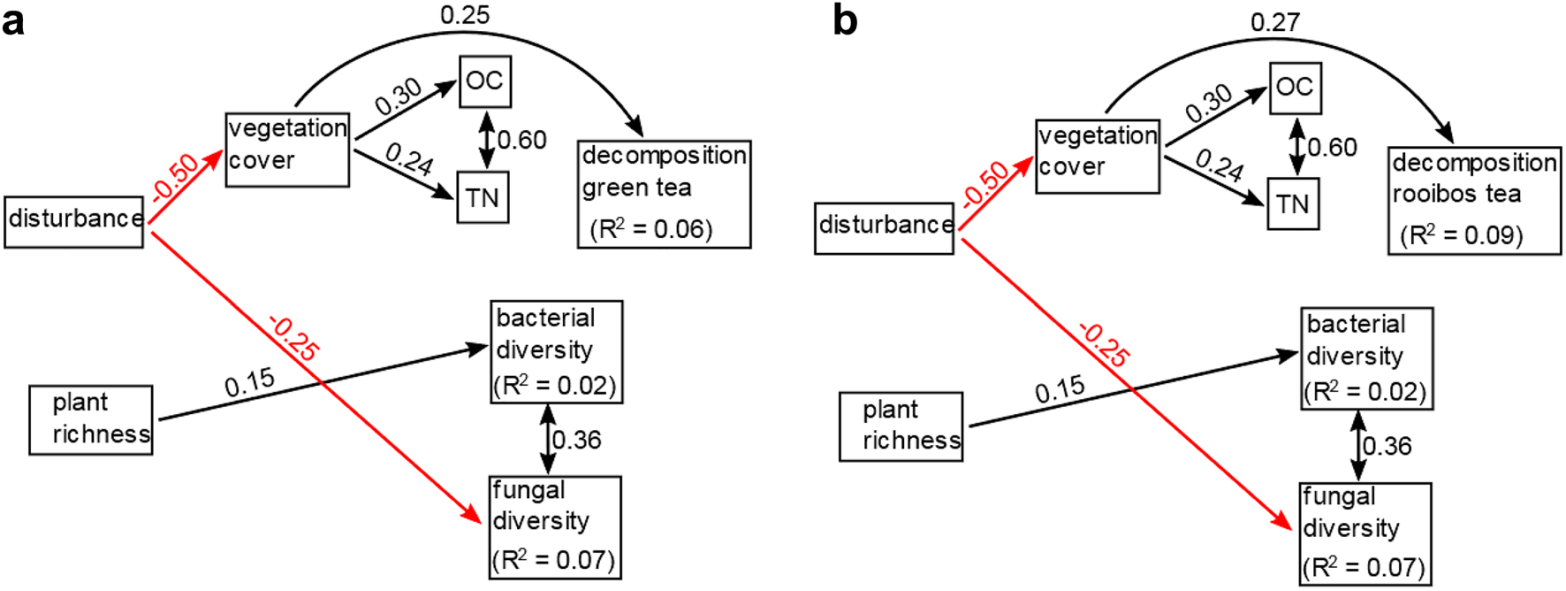
Structural equation models for the combination of sampling years 2016 + 2017. Subfigures show results for different soil functions as terminal response variable: decomposition of green tea (**a**), decomposition of rooibos tea (**b**). Arrows show direction of significant effects at *p* < 0.05, line color indicates sign of relationship (black: positive, red: negative). Double-headed arrows indicate bi-directional correlation. Standardized coefficients are given as numbers next to the arrows. Marginal R^2^-values of soil functions are given for microbial diversity and soil functions.

Across all models, soil disturbance had a significant negative effect on vegetation cover, with standardized coefficients ranging from − 0.42 to − 0.65 (Figs. 6, 7, 8). Additionally, soil OC and TN were correlated in all models (standardized coefficients 0.85, 0.44, and 0.60 for 2016, 2017, and 2016 + 2017, respectively). For 2017 data and for the combined data of both years, increasing vegetation cover significantly increased soil OC and TN content (Figs. 7 and 8), while for 2016 data this effect was not significant.

### Microbial diversity

Including the explained variation of fixed and random effects (vineyard, year), the SEM explained 63–67% of variation of bacterial diversity and 9–37% of variation of fungal diversity (Supplementary Table S4). Fixed effects explained between 2 and 19% of variation of bacterial diversity and 7–10% of fungal diversity, with the 2016 model showing the highest values for both microbial groups (Figs. 6, 7, 8). Bacterial and fungal diversity showed a significant correlation with a correlation coefficient of 0.36 for the model for 2016 + 2017 (Fig. 8).

Bacterial diversity was increased by increasing disturbance (standardized coefficient: 0.28, *p*-value = 0.002) and increasing plant richness (0.28, *p* < 0.001) for 2016 (Fig. 6). For the combined data (2016 + 2017, Fig. 8), the positive effect of plant richness on bacterial diversity was reproduced (0.15, *p* = 0.01), while this was not the case for the positive effect of soil disturbance. For 2016 data, bacterial diversity was decreased by increasing TN (− 0.40, *p* = 0.02). For fungal diversity, we observed a negative effect of soil disturbance on fungal diversity, which was significant for the combined data of both years (− 0.25, *p* = 0.007, Fig. 8).

### Soil functions

The models derived from data of 2016 explained about 61% of variance of soil respiration (Supplementary Table S4), 22% of green tea decomposition and 32% (54%) of rooibos tea decomposition. For 2017, explained variances for decomposition variables were 11% for green tea and 15% for rooibos tea. For combined data of 2016 + 2017, explained variances for decomposition variables were 6% for green tea and 9% for rooibos tea.

Soil respiration significantly increased with increasing disturbance (standardized coefficient: 0.25, *p* = 0.011, Fig. 6a) and soil TN (0.59, *p* < 0.001). Because of the strong correlation of soil OC and TN, we cannot discriminate between the effects of both variables on soil respiration.

Regarding the decomposition variables, we observed similar effects in both models explaining green tea and rooibos tea decomposition, but the size of the effects differed between years. For 2016 and the combined data set of both years, decomposition of both tea types was increased by increasing vegetation cover (Figs. 6b, c; 8a, b). Coefficients and p-values varied between tea types and years (coefficient for 2016, green tea: 0.33, *p* = 0.019, 2016: rooibos tea: 0.40, *p* = 0.004, 2016 + 2017, green tea: 0.25, *p* = 0.0013, rooibos tea = 0.27, *p* < 0.001). Because vegetation cover was negatively affected by disturbance consistently across years, this led to negative indirect effects of soil disturbance on decomposition of both tea types.

For 2016, we observed opposing effects of soil TN and OC on decomposition of rooibos tea. Total nitrogen had a negative effect on rooibos tea decomposition (− 0.38, *p* = 0.04, Fig. 6c), while soil OC had a positive effect with a similar magnitude (0.37, *p* = 0.03).. Given the collinearity of OC and TN for 2016 (correlation coefficient: 0.84), we cannot discriminate between both effects and conclude that these opposing effects level each other out, and thus, are not changing decomposition rates.

## Discussion

### Microbial diversity

#### Contrasting response of bacterial and fungal diversity to soil disturbance

Soil bacteria and fungi showed contrasting responses to soil management of vineyard inter-rows. While bacteria showed the lowest diversity in undisturbed complete cover inter-rows, fungi showed the lowest diversity in tillage inter-rows. Structural equation modeling revealed that soil disturbance had a positive direct effect on bacterial diversity for 2016 data but a negative direct effect on fungal diversity for data obtained in both sampling years 2016 and 2017 if analyzed together.

Contrasting effects of soil disturbance on bacterial and fungal diversity has been shown in previous studies^12,22^, however, for bacterial diversity the patterns are inconsistent among studies^9,10^. Tillage events cause a mechanical impact on the soil environment imposing shear forces on soil aggregates. Hyphal growing fungi have been shown to be strongly inhibited due to the mechanical impact of tillage^1,23^. For bacterial communities, the consequences of soil mechanical disturbance are less clear.

Studies focusing on organisms besides soil bacteria and fungi showed that moderate levels of disturbances by soil management can be beneficial for biological diversity in vineyard ecosystems^21,24^. This phenomenon is discussed as the Intermediate Disturbance Hypothesis^25^. Similar to the observations found for plants, frequent application of tillage could have suppressed strong competitive bacterial taxa preventing them from dominating the soil bacterial community^22^. Simultaneously, recreation of new soil microhabitats provided niches for less competitive pioneer taxa. Studies that confirm the validity of the Intermediate Disturbance Hypothesis for microbes are scarce, but examples exist for bacterial communities in biocrusts^26^.

Changes of microbial diversity associated with soil disturbance is not only a consequence of the mechanical effects of tillage or similar techniques but also of changes of soil organic matter content, which is an important determinant of microbial communities^27^. Studies investigating changes of soil organic matter content following to grassland-cropland conversion report a very fast and exponential decline of soil OC and TN within the first year following tillage^28,29^. In our study, we found a significant effect of soil disturbance on soil organic matter variables (OC and TN) 2 years after first tillage (2017), which indicates a less strong response of soil organic matter in our study compared to the grassland-cropland conversion studies. Further, the decline of soil OC and TN did not lead to a significant change of diversity of soil microbial communities, probably due to the delayed response of bacterial and fungal communities to changes of soil organic matter.

Besides alpha diversity of soil microbial communities, soil treatment also alters the community composition of soil bacteria and fungi^13,30^. These effects are particularly pronounced in long-term experimental set-ups^12^. In a recent European study, we used parts of the soil microbial community data of this work for an investigation of the relative effects of soil disturbance and soil variables on microbial community composition in vineyards at larger geographic scales^31^. The results of this European investigation showed that, at larger geographic scales, the effect of soil disturbance is fairly small compared to soil variables and other region-specific variables.

### Soil total nitrogen negatively affected bacterial diversity

Bacterial diversity was negatively affected by soil total nitrogen content for the 2016 data. As shown by the model, TN was highly correlated with soil OC. Total nitrogen consists mainly of organic nitrogen, which is bound in soil organic matter. Because organic nitrogen, organic carbon and soil organic matter content are tightly linked both OC and TN should be interpreted as proxies for the soil organic matter status. To our knowledge, a linear relationship between bacterial diversity and soil organic matter or total nitrogen has not been found by published studies; in contrast to fungal diversity, which responded positively to soil organic matter at a continental scale^27^.

Regarding the role of soil nitrogen for soil bacterial diversity, bacterial richness and Shannon diversity responded negatively to high levels of inorganic nitrogen (ammonium, nitrate) in a grassland fertilization experiment in China^32^. High content of total nitrogen might be correlated with high concentrations of ammonium and nitrate due to utilization and conversion of nitrogen bound in soil organic matter by microorganisms. However, we cannot confirm this hypothesis because inorganic nitrogen was not measured in our experiment.

In our study, we also checked for the dependence of bacterial diversity on soil pH, since it is a well-established hypothesis that soil pH is the main driver among soil properties for bacterial diversity at both the continental and the farm scale^32,33^. Since we did not find any relationship between soil pH and bacterial diversity, we assume that the short pH-gradient (7.08–7.64) was not suitable to have significant effects on bacterial diversity in our experiment.

### Plant richness beneficial for bacterial diversity

Bacterial diversity showed a positive response to plant richness, while fungal diversity showed a positive but non-significant trend. Examining the soil treatment types, the herbal mixture cover showed on the highest median values for plant richness, soil bacterial and soil fungal diversity. A common hypothesis on plant diversity shaping soil microbial diversity assumes that bacterial diversity is enhanced by the heterogeneity of resources provided by plants (root exudates, leaf litter, dead roots) and by provisioning of heterogeneous soil habitats that is shaped by plant roots^34^. In our study, while the plant communities of ‘complete cover’ plots were rather simple, basically consisting of grass species, the application of seed mixtures for the ‘herbal mixture’ plots led to plant communities with diverse life forms including many legumes, perennial and annual forbs, and deep-rooting as well as shallow-rooting species. This heterogeneity of plant communities might have been the cause of the increase of soil microbial diversity.

While our data underpins the hypothesis that plant diversity supports below-ground microbial diversity, other studies carried out at natural sites did not confirm this hypothesis^35,36^. The mixed results were attributed to the fact that the strength of the effect of plant diversity on microbial diversity might be dependent on scale^36^, soil nutrient legacy effects^35^ and other plant community characteristics besides plant richness (e.g., evenness^37^).

### Soil functions

#### Soil respiration directly increased by soil disturbance

Soil respiration was highest in the tillage treatment and lowest in the permanent cover treatment in 2016. Supporting this, SEM revealed that soil respiration was increased by soil disturbance.

We hypothesize that the higher level of respiration in disturbed soils is a consequence of the induction of bacterial activity by supply of fresh plant material, which was incorporated by tillage in April. This ‘priming’ of bacterial activity by labile carbon sources led to a break-down of stable SOM carbon pools^38^. Accordingly, this priming effect might be enhanced in soils under tillage because fresh plant material, which has grown over winter, was mechanically incorporated into the soil. However, labile carbon sources are also provided by the rhizosphere of the vegetation cover in permanent cover plots^39^. Thus, the net effects of tillage as well as other factors controlling the priming effect are still under debate^40^.

Studies in long-term experimental vineyards reported that soil disturbance negatively affected soil respiration after 22 years of continuous soil management (tillage vs. no-till^6^). Here, decreased activity of microbes was explained by significant depletion of soil carbon and nitrogen and loss of aggregate due to long-term application of tillage. We would assume similar long-term responses of soil respiration due to soil organic matter loss, but we also assume that the adaption of soil microbial communities and its functioning needs more time than the duration of our study to adapt to changing soil organic matter status.

#### Soil disturbance negatively affects decomposition by reduction of vegetation cover

Under tillage, the decomposed fraction of tea was lower compared to the other treatments, although this pattern was only significant for the green tea representing a labile resource, but not for rooibos tea representing a recalcitrant carbon resource and was not reproduced by the data from 2017.

Applying SEM, we observed that less efficient decomposition under tillage is attributed to the positive causal link between vegetation cover and decomposition meaning that increasing disturbance led to a decrease of vegetation cover, and thus negatively affected decomposition rates. Interestingly, the causal dependencies of decomposition, being mediated by vegetation cover, differed from soil respiration, which showed a direct response to soil disturbance. Measurements of soil respiration was done in the lab under equal soil moisture and temperature conditions for a short period of 24 h. Litter decomposition was conducted in the field and covering 90 days during the main grapevine growing season. Thus, it can be considered as a proxy for soil microbial activity at environmental conditions.

The process of litter decomposition is dependent on moisture with an optimum at intermediate levels^41^. Phases of drought lead to fast desiccation of the upper soil layers which impairs activity of microbial decomposers and reduces the physical fragmentation of litter compounds^42^. Permanent above-ground vegetation under no-till conditions supports soil structuring and aggregation, which in turn reduces desiccation of upper soil layers^17,43^.

In our study, we could not show a direct effect of soil microbial diversity on soil ecosystem functions such as litter decomposition. Since decomposition is a complex process, which involves interaction of different bacterial and fungal functional guilds^44^, it is assumed to be higher in soils with higher microbial richness^45^. However, evidence from field studies is largely lacking. Direct effects of microbial diversity on soil ecosystem functions have been shown in a multifunctionality context by using structural equation modelling^20^, but not for litter decomposition as such. The Tea Bag Index approach applied in our study is a standardized litter bag method for estimating decomposition rates, which provides the opportunity to assess decomposition data combined with data on microbial diversity and community composition at large geographic scales and environmental gradients^46,47^.

## Conclusion

In this study, we investigated the effects of soil management on microbial diversity and soil functions using a balanced experimental design across nine vineyards in Germany for two consecutive years. Although patterns found in one year were less pronounced or insignificant in the other year, we showed that soil disturbance represented as frequency of tillage events had direct effects on bacterial and fungal diversity. While fungi were negatively affected by disturbance, probably due to mechanical disruption of hyphal networks, bacterial diversity was enhanced by disturbance. For viticulture, this highlights the necessity to take into account that soil management options could favor specific groups of organisms while others are impaired.

Additionally, we could show that soil bacterial diversity is promoted by above ground plant richness in vineyards. Soil management that includes sowing of species-rich seed-mixtures might therefore not only be beneficial for diversity of pollinators and other insects, but also for belowground microbial diversity.

Occasional disturbance while maintaining a sufficient vegetation cover and a high plant diversity by application of seed-mixtures could be beneficial for soil microbial diversity and for supporting ecosystem functions in vineyards. However, variation of results between years requires multi-year, continuous sampling in order to derive recommendations for winegrowers.

## Methods

### Experimental sites and soil management variables

The study was carried out from 2015 to 2017 in nine vineyards in the wine-growing region Rhinehesse in the federal state Rhineland-Palatine, Germany (Fig. 1a and b, Supplementary Material Table S5). All vineyards selected for the study was planted at least 8 years before the start of the study and exhibited a cane-trained growing system with non-cropped inter-rows between grapevine rows. Grapevine cultivar varied between years, but were the the same within each vineyard (except one, see Supplementary Material Table S5). All grapevine were grafted onto rootstocks.. All vineyards were commercially used, agrochemical products were applied according to the customs of the vine-growers and following the local practice guidelines for integrated viticulture.

Prior to establishing experimental plots, the inter-rows of all vineyards were covered with grass-dominated vegetation for at least 6 years. Based on this initial state, 4 experimental plots each with a width of 6 inter-rows (with an inter-row space of approx. 2 m and a row-length of 20 m) were installed within each vineyard representing 4 soil treatment types (‘treatments’), which are characterized as follows (Fig. 1c):

- Tillage (ti): Vegetation was removed in all inter-rows by mechanical tillage twice a year (April and July) throughout the study period.
- Alternating tillage (at): Tillage in every second inter-row twice a year (April and July), while in every other inter-row mulching was applied. Tilled and non-tilled inter-rows were changed within plots every year.
- Herbal mixture (hm): Inter-rows were tilled at the beginning of the study and prepared for manual seeding of an herbal seed mixture. The seed mixture consisted of 25 herbal species (Supplementary Material Table S6). Sowing was only done once at the beginning of the study and the developing vegetation cover was managed by mulching throughout the study period.
- Complete cover (cc): Vegetation of all inter-rows (covered with grass-dominated vegetation) was managed by regular mulching depending on regrowth, similar to the initial management.

The treatments also reflected a soil disturbance gradient that is represented by tillage frequency, which we defined as the number of tillage actions per inter-row during the study period. While in the ‘cc’ treatment no tillage was done (representing the low soil disturbance along the gradient), the ‘ti’ treatment was treated by the maximum number of tillage actions (representing the maximum disturbance). The other treatments could be ordered by the number of tillage actions (tillage frequency) as follows, beginning from the highest disturbance: ‘ti’ > ‘at’ > ‘hm’ > ‘cc’ (Supplementary Fig. S1).

Additionally, to soil disturbance, the number of plant species was directly manipulated by inclusion of the ‘hm’-treatment. Thus, we created a variation of plant species richness between plots at each site, which is not confounded by soil disturbance.

### Vegetation sampling

Vegetation was assessed twice each in 2016 and 2017 (2016: Apr 4–7 and Oct 12–16; 2017: Apr 5–10 and Sep 18–21). In each plot, the two central inter-rows were surveyed within a rectangle of one square meter (0.5 × 2 m) avoiding the first and last 5 m of the inter-rows. Species identity and total vegetation cover per rectangle were recorded. For analysis, parameters of the two assessments per year were averaged. These mean values are assumed to represent a general estimation of vegetation cover and plant diversity for each year.

### Soil sampling

Soil samples were taken in June 2016 and 2017 during the period of grapevine flowering and about 8 weeks after the tillage of ‘ti’ and ‘at’ treatments (Supplementary Fig. S1). Eight subsamples were taken in the two central inter-rows of each plot to a depth of 10 cm and were pooled afterwards into one mixed sample, summing up to 72 samples per year. The field-moist soil was sieved using a mesh size of 2 mm to remove stones and plant particles. An aliquot of 6 g per soil sample was stored at − 20 °C until DNA extraction. In 2016, an aliquot of about 50 g soil was shipped on cooling elements to a laboratory in Fribourg, Switzerland, for analysis of soil respiration (see below). The remaining sample was used for the analysis of soil physicochemical parameters.

### Soil physicochemical parameters

Samples were analyzed using standard procedures as described in Schaller, 1991 ^48^. Soil pH was measured by suspension of soil samples in 0.01 M CaCl_2_-solution (1:1.5). The proportion of fine soil organic carbon (OC) and total nitrogen (TN) were determined following the Dumas combustion method and using a “Vario MAX CNS” analyzer (Elementar Analysensysteme GmbH, Langenselbold, Germany). To determine the content of OC for calcareous soils (pH > 6.9), the calcium-carbonate fraction was determined using the *Scheibler* method and subtracted, as inorganic C, from the carbon content.

### Microbial diversity

DNA was extracted from 0.25 g of mixed soil sample per inter-row by using the DNeasyPowerSoil Kit® following the manufacturer’s protocol (QIAGEN N.V., Venlo, Netherlands).

DNA sequencing was conducted for the bacterial V4 region of the 16S rDNA using the primers 515f.: 5’-GTGYCAGCMGCCGCGGTAA-3’ and 806rB: 5’-GGACTACNVGGGTWTCTAAT-3’^49^, and for the fungal Internal transcribed spacer ITS2 using the primer pair ITS4: 5’-TCCTCCGCTTATTGATATGC-3’ and fITS7: 5’-GTGARTCATCGAATCTTTG-3’^50,51^. Sequencing of 250 bp paired-end amplicons was conducted on a MiSeq Illumina machine at Génome Quebec Innovation Centre (Montreal, Canada).

Raw Illumina fastq reads were quality controlled with FastQC^52^, generally showing good quality. The reads were cleaned and filtered using sickle^53^.

For Bacteria, the software package Mothur (version 1.39.5) was used for sequence analysis^54^ while following the Standard Operating Procedure outlined on http://www.mothur.org/wiki/MiSeq_SOP. Briefly, the overlapping paired-end reads were combined using make.contig. Then, each unique sequence was aligned with align.seqs to the SILVA reference alignment release 132^55^. A distance matrix was calculated allowing for four mismatches. Chimeric sequences were identified using chimera.uchime and removed. Sequences matching ”Chloroplast-Mitochondria-unknown-Archaea-Eukaryota “ were also removed. Next, sequences were clustered using the opticlust clustering algorithm^56^ to build operational taxonomic units (OTUs) at a dissimilarity of 0.03. The resulting file was parsed to separate the data for each sample. OTUs were assigned a taxonomic group with classify.seqs using the RDP reference file release 11^57^ and a cut-off of 80% of the bootstrap value. Mothur was used to convert data into biom format files.

Sequence analysis for fungi was conducted using the software package PIPITS (version 1.3.x,^58^). This was necessary because Mothur relies on alignment of all sequences, which is not possible for fungi. PIPITS generates a biom file for OTU with the UNITE fungal ITS reference set based on the RDP classifier^59^.

Downstream sequence preprocessing was done in R version 3.4.2^60^ with the package phyloseq^61^. Unassignable sequences not belonging to the kingdom of bacteria or fungi were removed.

Shannon diversity Indices (H’) for bacteria and fungi were calculated from raw OTU data without rarefaction as well as subsampled OTU data to even sequencing depth using rarefaction. We observed that H’ from unrarefied and rarefied data were highly correlated (Pearson’s r = 0.98 for bacteria, and 0.99 for fungi, Supplementary Fig. 2). Hence we decided to use H’ from the raw OTU data without rarefaction following Haegeman et al., 2013 ^62^.Shannon diversity indices are referred to bacterial and fungal diversity, hereafter.

### Soil respiration and decomposition

Measurements of microbial respiration rates were carried out at the University Fribourg, Switzerland, using a micro-respirometer^63^. Soil samples from each inter-row (4.5 g) were moisturized to water saturation and measured with a micro-respirometer for about 22 h. Values are reported as the average consumption of oxygen (µg O_2_/ h * 1 g dry soil) between hours 10 and 20. Because measurements were done about 8 weeks after tillage and with soil samples in the laboratory, our data rather represent a response of microbes to the general characteristics of the soil including nutrient status and soil structure. For litter decomposition, we adopted the Teabag Index method^47^ by using two types of commercial tea (Lipton green tea, EAN: 87 22,700 05,552 5 and Lipton rooibos tea, EAN: 87 22,700 18,843 8) as standardized litter sample. Green tea, with a high fraction of hydrolysable compounds, decomposes more rapidly than rooibos tea^47^. Therefore, the use of these tea types allows discriminating between decomposition rates of labile organic material (green tea) and recalcitrant material (rooibos tea). Five teabag pairs composed of both types were buried 8–10 cm deep into the soil in each of the two central inter-rows per each plot in May 2016 and 2017. After in-situ incubation time of 90 days, teabags were recovered and the decomposed fraction of the starting weight was calculated for both tea substrates. We used the decomposed fraction of green tea and rooibos tea as response variable, rather than calculating the decomposition rate r and stabilization factor S, because we did not conduct preliminary analysis whether the premises for r and S made in Keuskamp et al., 2013^47^ applied to our site conditions^64^.

### Statistical analysis

Data analysis was carried out using two different approaches. First, the effects of soil treatment on all variables were analyzed using linear mixed models, analysis of variance (ANOVA) and pairwise comparison of treatments using Tukey contrasts. Secondly, structural equation models (SEM) were applied to unravel direct and indirect effects of soil disturbance, vegetation and soil variables on microbial diversity and soil functions. All analysis were carried out using R version 3.6.2^60^.

#### Effects of soil treatment types

Soil functions (decomposition of green tea and roobis tea, soil respiration), bacterial and fungal diversity, soil variables (OC, TN), vegetation cover and plant species richness were used as response to soil treatment in linear mixed models separately for both sampling years (package *nlme*,^65^). Since plant species richness was manipulated via sowing of a seed mixture, the ‘hm’ treatment was excluded in models using plant richness as response variable. Vineyard identity was included as random intercept to account for spatial correlation of samples at one site. We did not observe relevant deviations from homoscedasticity and normality of the residuals. Models were tested for significance using ANOVA; marginal means of response variables for each treatment were tested using Tukey contrasts (package *emmeans*,^66^).

#### Structural equation modeling (SEM)

We used SEM including variables on soil disturbance, soil variables and vegetation, microbial diversity, and soil functions. Structural equation models were fitted using piecewise SEM (package *piecewiseSEM*,^67^). The piecewise SEM approach uses local estimation of pathways within the model instead of global estimation based on the variance/covariance matrix of the underlying data^68^. An essential of piecewise SEM is its capability to include generalized linear mixed models, which is essential for our experimental design.

Structural equation modeling requires an a priori theoretical path model based on informed hypotheses about causal relationships between variables, which we compiled based on previous literature (Fig. 5 and Supplementary Table S2). Models were built based on the meta model using soil respiration, decomposition of green tea, and decomposition of rooibos tea as terminal response variable for each sampling year (2016 and 2017) and for both years combined. Because soil respiration was only measured in 2016, this response variable was only used for the 2016 data. All models included seven predictor and/or intermediate variables: disturbance, plant richness, vegetation cover, TN, OC, bacterial diversity, and fungal diversity. We allowed for bidirectional relationships between soil TN and OC as well as between bacterial and fungal diversity for all models, because data exploration showed indications of strong correlation between these variables. To account for spatial and temporal dependence we included random effect terms using vineyard (1|vineyard) and vineyard within year (1|year/vineyard) as random effects on the intercept for the single year models and for both sampling years combined, respectively.

Each model was analyzed by fitting each component model as a linear mixed-effect model (using the *nlme* package) and checking model validity by examining the residuals for normality and heteroscedasticity. Then, component models were assembled and a Fisher’s C test for conditional independency was applied to assure that no significant pathways were missing in the model^69^. Model parameters including coefficients, standard errors, test statistics, p-values, standardized coefficients, and R-squared values were retrieved from model summary tables.

## Supplementary Information

Supplementary Information 1.

Supplementary Table S3.

## Data Availability

The datasets generated during the study are made available at the PANGEA repository under the link https://doi.pangaea.de/10.1594/PANGAEA.948046 . Sequencing data are stored at the European Nucleotide Archive (ENA) retrievable with the primary accession number PRJEB31962 (http://www.ebi.ac.uk/ena/data/view/PRJEB31962).
