# Author Correction: Disentangling the mixed effects of soil management on microbial diversity and soil functions: A case study in vineyards

**DOI:** 10.1038/s41598-023-33154-7

**Published:** 2023-04-12

**Authors:** Martin Pingel, Annette Reineke, Ilona Leyer

**Affiliations:** 1 Department of Applied Ecology, Geisenheim University, Von-Lade-Str. 1, 65366 Geisenheim, Germany; 2 Department of Crop Protection, Geisenheim University, Von-Lade-Str. 1, 65366 Geisenheim, Germany

Correction to: *Scientific Reports* 10.1038/s41598-023-30338-z, published online 02 March 2023

The original version of this Article contained errors in the names of the authors Martin Pingel, Annette Reineke and Ilona Leyer, which were incorrectly given as Pingel Martin, Reineke Annette and Leyer Ilona.

The original Article and its accompanying Supplementary Information 1 have been corrected.

