# Supplementary material for: Disentangling the mixed effects of soil management on microbial diversity and soil functions: A case study in vineyards
Source: Sci Rep. 2023 Mar 2;13:3568. doi: 10.1038/s41598-023-30338-z (PMC9981623; doi:10.1038/s41598-023-30338-z)
Supplement: Supplementary file 1 — Supplementary Information 1. [file 41598_2023_30338_MOESM1_ESM.pdf]

Disentangling the mixed effects of soil management on microbial diversity and soil functions: A case study in vineyards

Supplementary Material

Martin Pingel*[1]; Annette Reineke[2]; Ilona Leyer[1]

[1]Geisenheim University, Department of Applied Ecology, Von-Lade-Str. 1, D-65366, Geisenheim, Germany

[2]Geisenheim University, Department of Crop Protection, Von-Lade-Str. 1, D-65366, Geisenheim, Germany

*Corresponding author: martin.pingel@hs-gm.de

Supplementary Table S1 Results of ANOVA of fitted linear models of soil functions, microbial diversity (Shannon-Wiener Index H'), soil variables (OC: organic carbon, TN: total nitrogen) and vegetation cover responding to treatment (included as a factor). ANOVA based on Wald tests fitted with 'lme' of the nlme package (significance levels: $p < 0.05$ (*), $p < 0.01$ (**), $p < 0.001$ (***)).

Response	Year	F-Value	p-Value	
bacterial H'	2016	5.92	0.0013	**
	2017	0.58	0.6304	
fungal H'	2016	1.34	0.2697	
	2017	4.24	0.0088	**
respiration	2016	3.56	0.0194	*
decomposition green tea	2016	7.03	0.0001	***
	2017	0.04	0.9892	
decomposition rooibos tea	2016	2.42	0.0748	
	2017	1.52	0.2172	
vegetation cover	2016	12.55	0.0001	***
	2017	28.19	0.0001	***
plant species richness	2016	7.09	0.0022	**
	2017	14.88	0.0001	***
OC	2016	0.39	0.7608	
	2017	0.94	0.4277	
TN	2016	0.50	0.6861	
	2017	0.60	0.6184	

Supplementary Table S2 Causal relationships between variables of the meta model including examples from literature supporting either positive (+) or negative (-) effects of the predictor on the respective response. Numbers refer to the graphical depiction of the meta model (Fig. 5). OC: soil organic carbon, TN: soil total nitrogen.

Number	Predictor	Response	Example references	sign of coefficient
1	disturbance	vegetation cover	1,2	-
2	disturbance	OC + TN	2–5	-
3	disturbance	bacterial diversity	6	-
	disturbance	bacterial diversity	2,7	+
	disturbance	fungal diversity	8,9	-
4	disturbance	soil respiration	3	-
	disturbance	soil respiration	10	-
	disturbance	litter decomposition	11,12	-
5	plant richness	OC + TN	2,13	+
6	plant richness	bacterial diversity	14	+
	plant richness	bacterial diversity	15,16	+/-
	plant richness	fungal diversity	17	+
7	plant richness	soil respiration	12	+
	plant richness	decomposition	12,18	+
8	vegetation cover	OC + TN	19,20	+
9	vegetation cover	bacterial diversity	21	+
	vegetation cover	fungal diversity	21,22	+
10	vegetation cover	soil respiration	23	+/-
	vegetation cover	decomposition	no examples found	+/-
11	OC + TN	bacterial diversity	20	+
	OC + TN	fungal diversity	20	+
12	OC + TN	soil respiration	24,25	+
	OC + TN	decomposition	25,26	+
13	bacterial diversity	soil respiration	27,28	+
	fungal diversity	soil respiration	29,30	+
	fungal diversity	soil respiration	28	-
14	bacterial diversity	decomposition	29,31	+
	fungal diversity	decomposition	29,31	+

References to Supplementary Table S2

1. Fiera, C. *et al.* Effects of vineyard inter-row management on the diversity and abundance of plants and surface-dwelling invertebrates in Central Romania. *Journal of insect conservation* **24,** 175–185 (2020).
2. Pingel, M., Reineke, A. & Leyer, I. A 30-years vineyard trial: Plant communities, soil microbial communities and litter decomposition respond more to soil treatment than to N fertilization. *Agriculture, Ecosystems & Environment* **272,** 114–125 (2019).
3. Belmonte, S. A. *et al.* Effect of Long-Term Soil Management on the Mutual Interaction Among Soil Organic Matter, Microbial Activity and Aggregate Stability in a Vineyard. *Pedosphere* **28,** 288–298 (2018).
4. Cotton, J. & Acosta-Martínez, V. Intensive Tillage Converting Grassland to Cropland Immediately Reduces Soil Microbial Community Size and Organic Carbon. *Agric. environ. lett.* **3,** 180047 (2018).
5. Poeplau, C. *et al.* Temporal dynamics of soil organic carbon after land-use change in the temperate zone - carbon response functions as a model approach. *Global change biology* **17,** 2415–2427 (2011).
6. Constancias, F. *et al.* Microscale evidence for a high decrease of soil bacterial density and diversity by cropping. *Agron. Sustain. Dev.* **34,** 831–840 (2014).
7. Lienhard, P. *et al.* Pyrosequencing evidences the impact of cropping on soil bacterial and fungal diversity in Laos tropical grassland. *Agron. Sustain. Dev.* **34,** 525–533 (2014).
8. Cho, H., Kim, M., Tripathi, B. & Adams, J. Changes in Soil Fungal Community Structure with Increasing Disturbance Frequency. *Microbial ecology* **74,** 62–77 (2017).
9. Schnoor, T. K., Lekberg, Y., Rosendahl, S. & Olsson, P. A. Mechanical soil disturbance as a determinant of arbuscular mycorrhizal fungal communities in semi-natural grassland. *Mycorrhiza* **21,** 211–220 (2011).
10. Pascault, N. *et al.* In situ dynamics of microbial communities during decomposition of wheat, rape, and alfalfa residues. *Microbial ecology* **60,** 816–828 (2010).
11. Faust, S., Koch, H.-J., Dyckmans, J. & Joergensen, R. G. Response of maize leaf decomposition in litterbags and soil bags to different tillage intensities in a long-term field trial. *Applied Soil Ecology* **141,** 38–44 (2019).
12. Tresch, S. *et al.* Litter decomposition driven by soil fauna, plant diversity and soil management in urban gardens. *The Science of the total environment* **658,** 1614–1629 (2019).
13. Lange, M. *et al.* Plant diversity increases soil microbial activity and soil carbon storage. *Nature communications* **6,** 6707 (2015).
14. Eisenhauer, N. Plant diversity effects on soil microorganisms: Spatial and temporal heterogeneity of plant inputs increase soil biodiversity. *Pedobiologia* **59,** 175–177 (2016).
15. Porazinska, D. L. *et al.* Plant diversity and density predict belowground diversity and function in an early successional alpine ecosystem. *Ecology* **99,** 1942–1952 (2018).
16. Prober, S. M. *et al.* Plant diversity predicts beta but not alpha diversity of soil microbes across grasslands worldwide. *Ecology letters* **18,** 85–95 (2015).
17. Shen, C., Wang, J., He, J.-Z., Yu, F.-H. & Ge, Y. Plant Diversity Enhances Soil Fungal Diversity and Microbial Resistance to Plant Invasion. *Applied and environmental microbiology* **87** (2021).
18. Weisser, W. W. *et al.* Biodiversity effects on ecosystem functioning in a 15-year grassland experiment: Patterns, mechanisms, and open questions. *Basic and Applied Ecology* **23,** 1–73 (2017).

19. García-Díaz, A., Marqués, M. J., Sastre, B. & Bienes, R. Labile and stable soil organic carbon and physical improvements using groundcovers in vineyards from central Spain. *The Science of the total environment* **621,** 387–397 (2018).
20. Maestre, F. T. *et al.* Increasing aridity reduces soil microbial diversity and abundance in global drylands. *Proceedings of the National Academy of Sciences of the United States of America* **112,** 15684–15689 (2015).
21. Vukicevich, E., Lowery, T., Bowen, P., Úrbez-Torres, J. R. & Hart, M. Cover crops to increase soil microbial diversity and mitigate decline in perennial agriculture. A review. *Agron. Sustain. Dev.* **36** (2016).
22. Cline, L. C. *et al.* Resource availability underlies the plant-fungal diversity relationship in a grassland ecosystem. *Ecology* **99,** 204–216 (2018).
23. Raich, J. W. & Tufekcioglu, A. Vegetation and soil respiration: Correlations and controls. *Biogeochemistry* **48,** 71–90 (2000).
24. Belmonte, S. A. *et al.* Effects of permanent grass versus tillage on aggregation and organic matter dynamics in a poorly developed vineyard soil. *Soil Res.* **54,** 797 (2016).
25. Curiel Yuste, J. *et al.* Microbial soil respiration and its dependency on carbon inputs, soil temperature and moisture. *Global Change Biol* **13,** 2018–2035 (2007).
26. Delgado-Baquerizo, M., García-Palacios, P., Milla, R., Gallardo, A. & Maestre, F. T. Soil characteristics determine soil carbon and nitrogen availability during leaf litter decomposition regardless of litter quality. *Soil Biology and Biochemistry* **81,** 134–142 (2015).
27. Bell, T., Newman, J. A., Silverman, B. W., Turner, S. L. & Lilley, A. K. The contribution of species richness and composition to bacterial services. *Nature* **436,** 1157–1160 (2005).
28. Yang, C., Liu, N. & Zhang, Y. Soil aggregates regulate the impact of soil bacterial and fungal communities on soil respiration. *Geoderma* **337,** 444–452 (2019).
29. Delgado-Baquerizo, M. *et al.* Microbial diversity drives multifunctionality in terrestrial ecosystems. *Nature communications* **7,** 10541 (2016).
30. Liu, Y.-R. *et al.* New insights into the role of microbial community composition in driving soil respiration rates. *Soil Biology and Biochemistry* **118,** 35–41 (2018).
31. Bonanomi, G., Capodilupo, M., Incerti, G., Mazzoleni, S. & Scala, F. Litter quality and temperature modulate microbial diversity effects on decomposition in model experiments. *Community Ecology* **16,** 167–177 (2015).

Supplementary Table S3:

See Supplementary CSV file ‘Supplementary_Table_S3_psem_coefficients.csv’

Table of path coefficients of structural equation models. All coefficients of all separately analyzed models are given. Columns represent the following information. Data are separated by semicolon (‘;’).

Year: Year of data set used in the model (2016, 2017, 2016+2017).

Response Variable/ Predictor Variable: response and predictor variable of the respective univariate relationship (bacterial diversity (BACT_DIV), decomposition green tea (DEC_GREEN), decomposition rooibos tea (DEC_RED), soil disturbance (DISTURBANCE), fungal diversity (FUNG_DIV), soil organic carbon (OC), vegetation cover (PLA_COV), plant species richness (PLA_RICH), soil respiration (RESP), soil total nitrogen (TN). Double tilde (‘~~’) indicate bidirectional relationship.

Coefficient: unstandardized coefficient of the respective path.

Standard Error: standard error of the coefficient.

DF: degrees of freedom.

Crit.Value/: t-statistics.

P.Value: p-value of the respective path.

Std.Estimate: standardized coefficient.

Supplementary Table S4 R-squared values (marginal, conditional) of soil microbial diversity, soil respiration, and litter decomposition, soil variables (OC: organic carbon, TN: total nitrogen), and vegetation cover obtained from structural equation models. Analyses were done separately for years 2016 and 2017 and for data of both years combined except for soil respiration. Marginal R-squared values represent variances explained by all pathways pointing towards the response variable, conditional R-squared values represent variances explained by all pathways and the random effect of vineyard and vineyards nested in years (for 2016 + 2017).

Response variable	Year(s)	Marginal R^2	Conditional R^2
bacterial diversity	2016	0.19	0.67
	2017	0.06	0.63
	2016+2017	0.02	0.67
fungal diversity	2016	0.10	0.29
	2017	0.09	0.21
	2016+2017	0.07	0.37
respiration	2016	0.61	0.66
decomposition green tea	2016	0.22	0.52
	2017	0.11	0.67
	2016+2017	0.06	0.71
decomposition rooibos tea	2016	0.32	0.54
	2017	0.15	0.59
	2016+2017	0.09	0.69
OC	2016	0.03	0.27
	2017	0.21	0.43
	2016+2017	0.08	0.32
TN	2016	0.05	0.36
	2017	0.14	0.36
	2016+2017	0.06	0.37
vegetation cover	2016	0.18	0.62
	2017	0.42	0.59
	2016+2017	0.24	0.64

Supplementary Table S5 Details of vineyards used for the study.

ID	Latitude	Longitude	Grape cultivar	Year of planting
DE1	49.9455°N	7.9949 °E	Pinot noir	2002
DE2	49.9467 °N	8.0130 °E	Dakapo	2002
DE3	49.9349 °N	7.9730 °E	Pinot noir	2001
DE4	49.9254 °N	8.1146 °E	Mueller-Thurgau	1987
DE5	49.9284 °N	8.1031 °E	Pinot blanc	2007
DE6	49.8929 °N	8.0765 °E	Silvaner	1988
DE7	49.8212 °N	8.3080 °E	Dornfelder	1994
DE8	49.8320 °N	8.3486 °E	Riesling	1994
DE9	49.8550 °N	8.3545 °E	Not the same across all plots	1992

Supplementary Table S6 Plant species used for the seed mixture for the treatment herbal seed mixture cover ('hm'). All species were included in equal amounts.

Achillea millefolium	*Medicago lupulina*
Agrostemma githago	*Onobrychis viciifolia*
Anethum graveolens	*Papaver rhoeas*
Calendula officinalis	*Phacelia tanacetifolia*
Centaurea cyanus	*Plantago lanceolata*
Cichorium intybus	*Prunella vulgaris*
Consolida regalis	*Salvia pratensis*
Coriandrum sativum	*Sanguisorba minor*
Daucus carota	*Trifolium incarnatum*
Fagopyrum esculentum	*Trifolium repens*
Lotus corniculatus	*Trifolium subterraneum*
Malva sylvestris	*Viola arvensis*
Matricaria recutita	

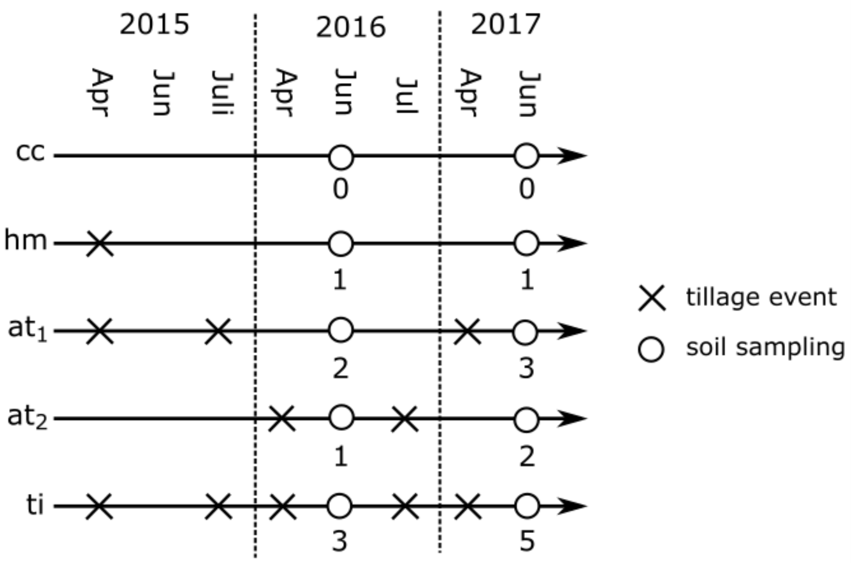

Supplementary Fig. S1 Tillage frequency as variable for disturbance intensity for the different treatments. Along the course of the study (2015 – 2017) the different vineyard plots experienced different numbers of tillage events (crosses along the time axis) dependent on the treatment (‘cc’: complete cover, ‘hm’: herbal mixture, ‘at’ alternating tillage, ‘ti’ tillage). At each time point indicated (white circles) soil sampling was conducted, the cumulative number of tillage events were recorded as a proxy for disturbance intensity for the respective soil sample (number below the white circles).

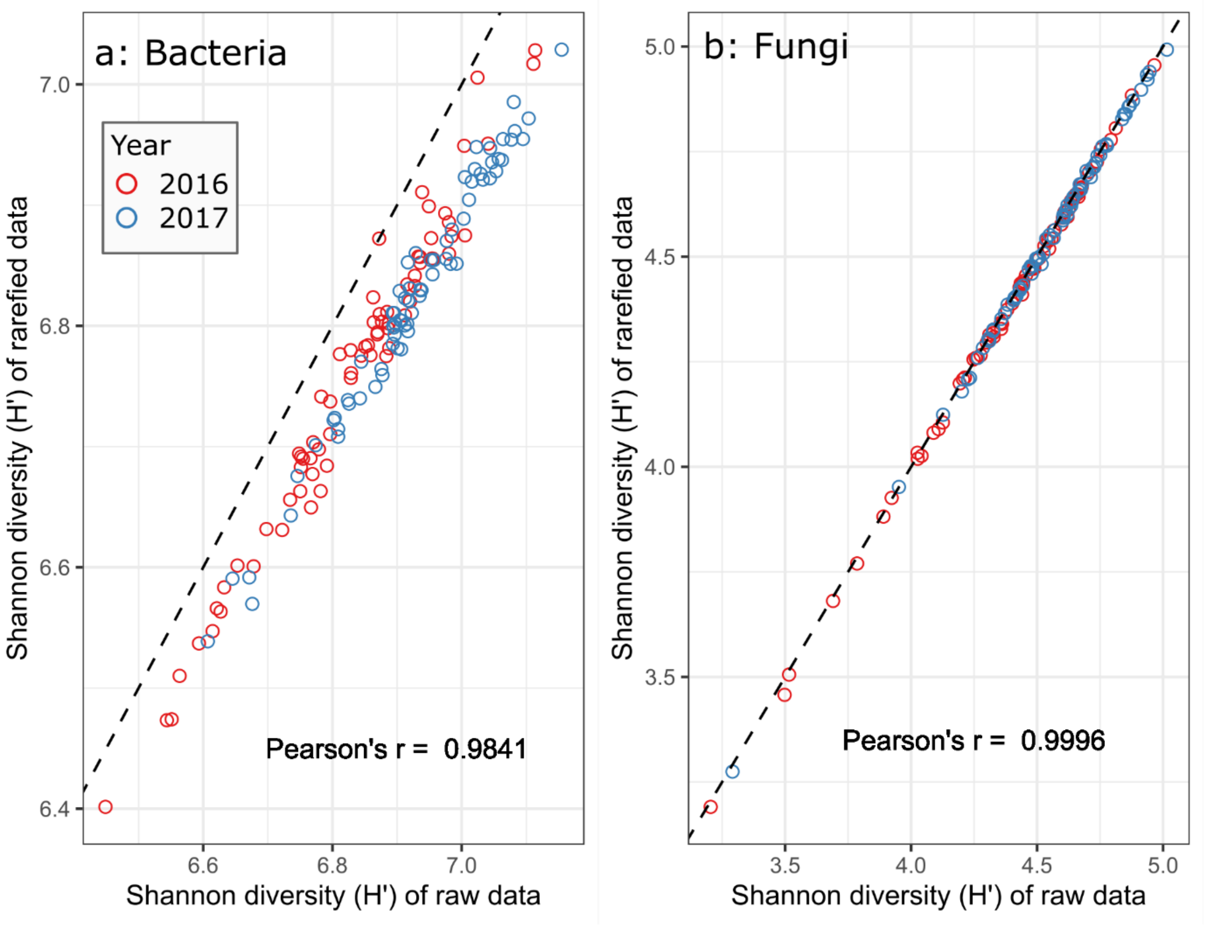

Supplementary Fig. S2 Correlation of Shannon Diversity indices (H') of raw OTU tables (x-axes) and rarefied OTU tables (y-axes) for bacteria (a) and fungi (b). Rarefaction was done by subsampling raw OTU tables to even sequencing depth across samples based on the lowest sequencing depth for each microbial group (bacteria = 13651, fungi = 30136). Dashed line represents the diagonal y = x; colours of circles represent year (red = 2016, blue = 2017).
